# Subclinical Myocardial Injury and Risk of COVID-19 in the General Population: The Trøndelag Health Study

**DOI:** 10.1093/clinchem/hvab267

**Published:** 2021-12-18

**Authors:** Magnus Nakrem Lyngbakken, Kristian Hveem, Helge Røsjø, Torbjørn Omland

**Affiliations:** 1 Department of Cardiology, Akershus University Hospital, Lørenskog, Norway; 2 Institute of Clinical Medicine, University of Oslo, Oslo, Norway; 3 HUNT Research Centre, Department of Public Health and General Practice, Norwegian University of Science and Technology, Levanger, Norway; 4 Levanger Hospital, Nord-Trøndelag Hospital Trust, Levanger, Norway; 5 Division of Research and Innovation, Akershus University Hospital, Lørenskog, Norway

**Keywords:** viral diseases, troponin, epidemiology studies, cardiac markers

## To the Editor:

Cardiovascular disease (CVD) is a risk factor for a severe clinical course in COVID-19 (1), and CVD risk factors associate with the risk of contracting COVID-19 in the general population (2). Subclinical myocardial injury, quantified by cardiac troponin, is common in patients hospitalized with COVID-19 (3), but its association with risk of COVID-19 is unknown. We hypothesized that subclinical myocardial injury is associated with incident COVID-19 in the general population.

The Trøndelag Health (HUNT) Study is the largest population-based cohort in Norway (4), and the fourth study visit (HUNT4) was conducted from 2017 to 2019 including 56 078 participants. The study was approved by the ethics committee and all participants provided informed written consent.

For the current investigation, we included all study participants from HUNT4 with measurement of high-sensitivity cardiac troponin I (cTnI, ARCHITECT STAT High Sensitive Troponin, assay 99th percentile 16 ng/L for women and 34 ng/L for men). cTnI analysis was performed within 24 h of serum collection. Information on incident COVID-19 was acquired from the Norwegian Surveillance System for Communicable Diseases and all-cause mortality from the Norwegian Cause of Death Registry. Clinical end points were obtained through May 31, 2021. We used a Fine and Gray proportional subhazards model to analyze associations with incident COVID-19, using all-cause mortality as competing risk. We adjusted for age, sex, and established risk factors of severe COVID-19 (i.e., CVD, diabetes mellitus, body mass index, and current smoking) (1). Prognostic accuracy was assessed by *c* statistics and the net reclassification index (NRI).

cTnI was measured in 37 835 study participants from HUNT4. During a median follow-up time of 1083 (interquartile range 943 to 1152) days, 237 events (0.6%) were registered for incident COVID-19 (including 4 hospital admissions) and 1030 (2.7%) events for all-cause mortality. No COVID-19 related deaths were registered. Study participants with incident COVID-19 were younger, less frequently current smokers, had less frequently established CVD, and lower concentrations of cTnI. Most of these differences were attenuated after adjustment for age and sex (Table 1). After adjustment for age and sex, the difference in cTnI between groups was 8.2% (95% CI, −2.9 to 19.4%). Lower concentrations of log-transformed cTnI were associated with incident COVID-19 (subdistribution hazard ratio [sHR] 0.77; 95% CI, 0.67–0.89). This association was no longer significant in adjusted analysis (adjusted sHR [asHR] 1.02; 95% CI, 0.87–1.20). The results were similar when limiting analysis to 2020, before the initiation of the Norwegian coronavirus immunization program (asHR 0.90; 95% CI, 0.66–1.22). There was no difference in the associations of cTnI with incident COVID-19 in study participants with (asHR 1.27; 95% CI, 0.72–2.25) or without established CVD (asHR 1.01; 95% CI, 0.86–1.19, *P* for interaction = 0.72). cTnI above the sex-specific 99th percentile (asHR 0.73, 95% CI, 0.18–2.95) or established CVD per se (asHR 0.77; 95% CI, 0.41–1.42) were not associated with incident COVID-19. cTnI did not improve the *c* statistics or NRI when added to a basic risk model constructed on age, sex, and established risk factors for severe COVID-19 (*c* index 0.686; 95% CI, 0.654–0.718, vs 0.686; 95% CI, 0.654–0.718, *P* for comparison = 0.97; NRI 0.087, 95% CI, −0.204 to 0.240). C-reactive protein (CRP) was not associated with incident COVID-19 (*c* index 0.514; 95% CI, 0.474–0.554), and there were no model improvements when cTnI was added to the basic risk model and CRP.

**Table:** Table 1. Baseline characteristics according to incident COVID-19.^a^

	Total cohort (n = 37 835)		No COVID-19 (n = 37 598)		Incident COVID-19 (n = 237)		*P*	
	n	Value	n	Value	n	Value	Unadjusted	Adjusted for age and sex
Male sex, n (%)	37 835	17 081 (45.1%)	37 598	16 974 (45.1%)	237	107 (45.1%)	>0.99	0.85
Age, years	37 835	55.4 (41.1–68.4)	37 598	55.5 (41.2–68.5)	237	43.8 (30.9–52.8)	<0.001	<0.001
Current smoking, n (%)	37 060	3228 (8.7%)	36 837	3219 (8.7%)	223	9 (4.0%)	0.012	0.034
Higher education, n (%)	37 001	13 817 (37.3%)	36 781	13 732 (37.3%)	220	85 (38.6%)	0.73	0.27
Medical history								
Diabetes mellitus, n (%)	36 816	2257 (6.1%)	36 594	2247 (6.1%)	222	10 (4.5%)	0.40	0.58
Angina pectoris, n (%)	35 543	1117 (3.1%)	35331	1112 (3.1%)	212	5 (2.4%)	0.69	0.32
Myocardial infarction, n (%)	35 733	1422 (4.0%)	35 520	1418 (4.0%)	213	4 (1.9%)	0.16	0.86
Heart failure, n (%)	35 486	654 (1.8%)	35 273	651 (1.8%)	213	3 (1.4%)	>0.99	0.22
Atrial fibrillation, n (%)	35 286	1884 (5.3%)	35 078	1879 (5.4%)	208	5 (2.4%)	0.06	0.66
Stroke, n (%)	35 532	1170 (3.3%)	35 319	1166 (3.3%)	213	4 (1.9%)	0.33	0.76
Any cardiovascular disease^b^, n (%)	37 835	4736 (12.5%)	37 598	4724 (12.6%)	237	12 (5.1%)	<0.001	0.36
Cancer, n (%)	35 795	2852 (8.0%)	35 585	2842 (8.0%)	210	10 (4.8%)	0.10	0.77
Antihypertensive therapy, n (%)	37 835	8739 (23.1%)	37 598	8715 (23.2%)	237	24 (10.1%)	<0.001	0.26
Lipid lowering therapy, n (%)	37 835	5994 (15.8%)	37 598	5979 (15.9%)	237	15 (6.3%)	<0.001	0.30
Body mass index, kg/m^2^	37 420	26.8 (24.0–30.0)	37 184	26.8 (24.0–30.0)	236	27.3 (24.1–30.9)	0.16	0.06
Waist-to-hip ratio	35 858	0.95 (0.90–1.01)	35 629	0.95 (0.90–1.01)	229	0.96 (0.89–1.02)	0.52	0.008
Heart rate, bpm^c^	36 319	71 (64–80)	36 088	71 (64–80)	231	71 (65–82)	0.21	0.37
Systolic blood pressure, mmHg	37 675	126 (115–139)	37 439	126 (115–139)	236	120 (110–132)	<0.001	0.28
Diastolic blood pressure, mmHg	37 675	72 (65–79)	37 439	72 (65–79)	236	70 (62–76)	<0.001	0.041
Total cholesterol, mg/dL	37 835	5.2 (4.4–6.0)	37 598	5.2 (4.4–6.0)	237	5.0 (4.2–5.7)	0.008	0.31
HDL^d^ cholesterol, mg/dL	37 835	1.3 (1.1–1.6)	37 598	1.3 (1.1–1.6)	237	1.3 (1.1–1.5)	0.004	0.005
Hb A_1c_, %	37 694	5.2 (5.0–5.5)	37 459	5.2 (5.0–5.5)	235	5.1 (4.9–5.4)	<0.001	0.64
Hemoglobin, g/dL	37 699	14.6 (13.7–15.5)	37 464	14.6 (13.7–15.5)	235	14.6 (13.6–15.7)	0.80	0.09
White blood cell count, 10^9^/L	37 697	6.7 (5.7–7.9)	37 462	6.7 (5.7–7.9)	235	6.5 (5.5–7.7)	0.07	0.16
eGFR^c^, mL/min/1.73m^2^	37 835	93.0 (80.0–105.0)	37 598	93.0 (80.0–105.0)	237	104.0 (89.0–117.0)	<0.001	0.48
CRP^c^, mg/L	37 835	1.3 (0.6–2.7)	37 598	1.3 (0.6–2.7)	237	1.3 (0.6–3.5)	0.45	0.007
CRP, mg/L (range)	37 835	0.1 to 160.0	37 598	0.1 to 160.0	237	0.1 to 77.0	NA	NA
Detectable cardiac troponin I^d^, n (%)	37 835	24 631 (65.1%)	37 598	24 508 (65.2%)	237	123 (51.9%)	<0.001	0.58
Cardiac troponin I^e^, ng/L	37 835	1.8 (0.6–3.5)	37 598	1.8 (0.6–3.5)	237	1.3 (0.6–2.6)	<0.001	0.15
Cardiac troponin I, ng/L (range)	37 835	0.6 to 1571.3	37 598	0.6 to 1571.3	237	0.6 to 74.6	NA	NA

a Data are reported as absolute numbers (proportion) or median (interquartile range) unless otherwise stated.

b Any cardiovascular disease = history of angina pectoris, myocardial infarction, heart failure, atrial fibrillation, and/or stroke.

c Abbreviations: bpm, beats per minute; eGFR, estimated glomerular filtration rate; CRP, C-reactive protein; NA, not applicable.

d HDL, high-density lipoprotein. To convert cholesterol concentrations from mg/dL to mmol/L, multiply by 0.02586.

e Detectable cardiac troponin I = above or at limit of detection (1.2 ng/L). Cardiac troponin I concentrations below the limit of detection were assigned a value of 0.6 ng/L.

In this population-based study with prospective measurement of cTnI, we found no association between subclinical myocardial injury, established CVD, and risk of incident COVID-19. Considering the established link between CVD, cardiac troponins, and COVID-19 severity (1), it is surprising that study participants with incident COVID-19 exhibited lower concentrations of cTnI. These study participants, however, were considerably younger, consistent with the demographic COVID-19 trends in Europe (5), and the absolute differences and prognostic properties of cTnI were attenuated in adjusted analyses.

Our study did not permit investigations of COVID-19 severity, as we acquired data on incident COVID-19 from the national registry of communicable diseases. The number of COVID-19 events was low, as Norway has been modestly affected by the ongoing pandemic. Severe acute respiratory syndrome coronavirus 2 (SARS-CoV-2) testing is performed on clinical indication and accordingly non-systematic, possibly underestimating the true number of COVID-19 patients. The number of hospital admissions was low, barring any meaningful analyses in this regard.

In conclusion, our study does not support the hypothesis of an association between subclinical myocardial injury and incident COVID-19 in predominantly healthy community dwellers. Populations with higher incidence of severe COVID-19 are needed to assess whether cTnI is an independent risk factor for hospital admission in COVID-19.

## Nonstandard Abbreviations

CVD, cardiovascular disease; HUNT Study, Trøndelag Health Study; cTnI, cardiac troponin I; asHR, adjusted subdistribution hazard ratio.

## Employment or Leadership

None declared.

## Consultant or Advisory Role

T. Omland, Roche Diagnostics, Abbott Diagnostics, and CardiNor.

## Stock Ownership

T. Omland, CardiNor.

## Honoraria

H. Røsjø, CardiNor, and SpinChip Diagnostics; T. Omland, Siemens Healthineers, Roche Diagnostics, and Abbott Diagnostics

## Expert Testimony

None declared.

## Patents

None declared.

## Other Remuneration

T. Omland, nonfinancial support from Novartis, Abbott Diagnostics, Roche Diagnostics, and SomaLogic not related to the current work.
